# Soil Disturbance as a Grassland Restoration Measure—Effects on Plant Species Composition and Plant Functional Traits

**DOI:** 10.1371/journal.pone.0123698

**Published:** 2015-04-13

**Authors:** Tim Schnoor, Hans Henrik Bruun, Pål Axel Olsson

**Affiliations:** 1 Biodiversity, Department of Biology, Lund University. The Ecology Building, SE-223 62 Lund, Sweden; 2 Department of Biology, University of Copenhagen, Universitetsparken 15, 2100 Copenhagen Ø, Denmark; USDA-ARS, UNITED STATES

## Abstract

Soil disturbance is recognized as an important driver of biodiversity in dry grasslands, and can therefore be implemented as a restoration measure. However, because community re-assembly following disturbance includes stochastic processes, a focus only on species richness or establishment success of particular species will not inform on how plant communities respond ecologically to disturbance. We therefore evaluated vegetation development following disturbance by quantifying species richness, species composition and functional trait composition. Degraded calcareous sandy grassland was subjected to experimental disturbance treatments (ploughing or rotavation), and the vegetation was surveyed during four subsequent years of succession. Treated plots were compared with control plots representing untreated grassland, as well as nearby plots characterized by plant communities representing the restoration target.

Species richness and functional diversity both increased in response to soil disturbance, and rotavation, but not ploughing, had a persistent positive effect on the occurrence of specialist species of calcareous sandy grassland. However, no type of soil disturbance caused the plant species composition to develop towards the target vegetation. The disturbance had an immediate and large impact on the vegetation, but the vegetation developed rapidly back towards the control sites. Plant functional composition analysis indicated that the treatments created habitats different both from control sites and target sites. Community-weighted mean Ellenberg indicator values suggested that the observed plant community response was at least partially due to an increase in nitrogen and water availability following disturbance. This study shows that a mild type of disturbance, such as rotavation, may be most successful in promoting specialist species in calcareous sandy grassland, but that further treatments are needed to reduce nutrient availability. We conclude that a functional trait based analysis provides additional information of the vegetation response and the abiotic conditions created, complementing the information from the species composition.

## Introduction

Reduction, cessation or suppression of disturbance, such as forest wildfires and dune dynamics, are reasons for a decline in species richness observed today in many of the World’s ecosystems [1, 2]. In managed landscapes, species adapted to disturbance may suffer negative population trends due to both too much and too little disturbance [3]. The decrease of many species in cultural landscapes [4] indicates that the management efforts are limited by our knowledge about the ecological factors and historical land-use that has shaped these habitats. Grazing continuity [5, 6] and low availability of certain macronutrients [7] are important and well-known drivers of grassland plant diversity, but it is increasingly recognized that soil disturbance and the occurrence of bare soil may act as limiting factors to threatened species [8, 9, 10]. Mechanical soil disturbance, such as soil perturbation and topsoil removal, could therefore function as a restoration measure [10, 11, 12, 13].

Restoration success is often based on the occurrence and positive population trends of threatened species. However, community assembly after a major perturbation is partly a deterministic filtering process based on functional traits [14] and partly a stochastic process based on propagule supply and dispersal [15]. The occurrence of species of conservation interest may be particularly stochastic and unpredictable [16]. In contrast, plant functional traits are likely to provide a more detailed indicator of the causes of restoration success or failure. The concept of ecosystem functioning within restoration ecology has mostly focused on how to restore ecosystem functions, such as productivity and nutrient accumulation [17]. In one of the few studies with a more theoretical functional basis, Pywell et al. [18] showed that specific traits are related to establishment success and proliferation during different time-stages in succession. Alternatively, Römermann et al. [19] showed that combining functional and floristic information helped in choosing between different management options in grazed grasslands. These studies show that using a functional approach can be important when trying to understand how to interpret the effects of restoration. Kleyer [20] found that high levels of disturbance favored annual plants and decreased age upon first reproduction, height, vertical biomass and lateral extension, and increased the potential for long-range dispersal. Grazing on the other hand increased the abundance of species that regenerate by means of a persistent seed bank and have a higher leaf toughness and leaf dry matter content [21, 22]. Grime [23] argued that traits in a system can either converge or diverge depending on the kind of disturbance, which may further impact community diversity.

In addition to analyzing individual plant functions as an indication of ecosystem proporties, functional diversity (FD) can be used to quantify this property of biodiversity [24, 25, 26]. Plant functional diversity has been shown to be important for invasion resistance [27], increase with grazing continuity [28] and can be an indication of various ecosystem services provided by a specific grassland [29].

A successful restoration is one where threatened species are favored and the species composition develops towards that of a reference site. In this study, we evaluated the effect of disturbance, implemented as a restoration option, on both species composition and functional composition in calcareous sandy grassland where the decline in threatened species is believed to be a consequence of reduced soil disturbance. We defined success as the development towards a species composition similar to plots with target vegetation or a general increase in threatened species, and failure as development towards untreated control plots. We also defined an overall increase in plant richness and functional diversity as a success because of the higher potential for such communities to support other groups of organisms [29]. We hypothesized that soil disturbance in grasslands with a historical land use with regular soil disturbance would lead to (1) increased proportion of specialist species, (2) increased taxonomic and functional diversity, (3) a taxonomic composition and functional composition more similar to target vegetation. We also investigated what the plant functional composition suggest about the mechanisms behind vegetation change in treated plots.

## Materials and Methods

### Site description

The experimental site is located at the Rinkaby military training ground (55°58N 14°18E) in eastern Scania, southernmost Sweden. The area has a mean annual precipitation of 500–550 mm per year, and a mean annual temperature of 7.5°C (based on data from 1956 to 2004). The field consists of 420 hectares Natura 2000 habitats [30]. The dominant vegetation on the study site closely resembles Fennoscandian lowland species-rich, dry to mesic grasslands (N6270, EU habitat directive 92/43/EEC) with native grasses such as *Festuca rubra*, *Helictotrichon pubescens* as well as the putatively introduced *Festuca brevipila*, and forbs such as *Medicago sativa* ssp. *falcata* and *Galium verum*. There are small areas of vegetation in the experimental area that can be characterized as “sand steppe” [31], classified as xeric sand calcareous grasslands (N6120, EU habitat directive 92/43/EEC), or Koelerio-glaucon type vegetation [32]. Typical species are *Koeleria glauca* and the endemic *Dianthus arenarius* ssp. *arenarius*; the former conventionally used as a ‘diagnostic species’ of the type.

Sand steppe is a threatened habitat with estimates of between only 20–55 ha remaining in Sweden [33, 34] and it is the restoration target in this study. It is also characterized by dry, infertile sandy soils low in nutrients and high in pH (>7.5). The site lies on calcareous bedrock, and the soil is dominated by sand, which has created the abiotic properties necessary for the formation of this rare habitat type. The decline in sand steppe area is most probably because of acidification, nitrogen deposition and abandonment of traditional agricultural practices [35, 36, 37]. The study area became a military training area in 1899, before which most of the area was part of a low-intensity ambulating farming system during the 18^th^ and 19^th^ centuries [30], with long periods of fallow and grazing (15–30 years) followed by one or two years with growing buckwheat and rye [34, 38]. Military exercises combined with grazing ensured that small-scale soil disturbance continued to occur frequently in the area for many years. Today, large parts of the area are grazed but with little mechanical soil disturbance.

### Experimental setup

In May 2006, a split-plot experiment consisting of three blocks was set up at Rinkaby [11]. Because these areas have been used for low-intensity agriculture historically, we used ploughing and rotavation as means of mimicking historical soil disturbance. Each of the blocks consisted of four replicates each of ploughed and rotavated plots, and eight control plots, thus a total of 48 plots. The ploughing and rotavation treatments were not mixed. The experiment was performed with permission from the landowner (The Swedish Armed Forces) and from The County Administrative Board in Skåne since the area is protected within the Natura 2000 framework. Rotavation crushed the sod of the grassland, mixing the topsoil layer (approximately 10 cm depth). Ploughing overturned the soil to a depth of 30 cm, leaving no visible clumps of sod. Four plots measuring 5 × 60 m were ploughed and four were rotavated, each with a control plot beside them in each block [11].

For the present study we selected one block characterized by high pH in all plots, and we included 4 control plots, 4 ploughed plots and 4 rotavated plots, thereby limiting this study to 12 plots. In order to limit the present study, we randomly selected 4 out of the 8 control plots. Soil samples taken one week after the treatments showed that pH (H_2_O) varied between 7.4 and 8.2, but with no significant effect of the disturbances. There was a small increase in extractable phosphorous in the rotavated plots but no effect of ploughing [11]. In addition to the treatment plots, we established two plots in areas with target vegetation.

### Vegetation survey

The 8 treatment plots and the 4 control plots were surveyed during four summers, from 2007 until 2010, and the target plots were surveyed in 2008 and 2010. The surveys took place during late June or early July to enable recording as many spring and summer species as possible. In each plot, ten squares measuring 0.25 m^2^ were inventoried using a pinpoint frame with ten pins per square. The squares were placed on a line evenly distributed within each plot. We recorded the number of times each species touched the pins, yielding a non-destructive abundance measure with a demonstrated correlation with aboveground biomass [39].

One restoration goal was to achieve colonization of the specialist plant species characteristic of the sand steppe habitat as well as nationally red-listed species. To evaluate this we used lists of specialist species from other studies in the same area [13] and the national red-list according to [40], in addition to the target plots. We also included the bryophyte *Syntrichia ruraliformis* as a sand steppe specialist [35].

### Trait collection

Traits were collected empirically in each of the three treatments. Collection took place in June 2007 and 2008 and followed the standardized protocol of Cornelissen et al. [41]. Ten individuals (three from ploughed plots, three from rotavated plots and four from control plots) of each of the 17 species were collected, and placed in water in a cooling box immediately after cutting. Thereafter specific Leaf Area (SLA), Leaf Dry Matter Content (LDMC), leaf tensile strength, canopy height and reproductive height were measured for each plant. Leaf traits were measured 24 hours after collection, thereby measuring all leaf traits on fully hydrated leaves. The mean leaf trait values from these ten individuals were used. Finally, for plant canopy height we used 25 individuals (eight from ploughed, eight from rotavated and nine from control plots). The same mean trait value was used for each species across treatments, and thus ignoring intraspecific variation. When studying the values for the different traits in each of the treatments, we found no or only small differences in mean values.

In addition to canopy height and the above-mentioned leaf traits, mycorrhizal status, seed mass and Raunkiær life form were retrieved from databases (Table 1). The hypothesized relationships between these traits and their ecosystem functions are summarized in Table 1. LDMC is related to SLA but may not capture exactly the same functions [41, 42]. Leaf tensile strength is the force needed to tear a leaf apart divided by the width of the leaf. Tensile strength is a good indicator of carbon investment in leaves, contributes to leaf lifespan [41] and resistance of a leaf against mechanical damage. These three leaf traits are similar, but not identical. Using all three was considered appropriate for assessing community response to disturbance. Together these are related to relative growth rate, phenotypic plasticity, stress tolerance and leaf longevity [43].

**Table 1:** The measured functions and a short description of which functions they are related to.

**Plant attribute**	**Functions**	**Reference to function**
SLA (mm^2^ mg^-1^) ^a^	Relative growth rate, photosynthetic rate	[41, 64, 65]
LDMC (mg g^-1^) ^a^	Relative growth rate, palatability	[42, 64, 65]
Tensile strength (g mm^-1^) ^a^	Leaf lifespan	[41]
Plant height (mm) ^a^	Competitive ability	[41, 43]
Reproductive height (mm) ^a^	Wind dispersal, animal dispersal	[66, 67]
Seed mass (g seed^-1^)^b^	Dispersal, seed bank longevity, establishment success	[43, 68]
Mycorrhizal status (categorical) ^c^	Phosphorus uptake	[69]
Raunkiær life form (categorical)^d^	Disturbance/stress adaptation	[70, 71]

Footnotes indicate from where the trait data used in the study was collected.

^a^ Collected at site

^b^ [72]

^c^ [73]

^d^LEDA traitbase [74]

As a complement to the functional trait values, we used Ellenberg indicator values for light (L), pH (R) and soil nutrient (N) preferences [44]. We used the values from Ellenberg et al. [44], supplemented with Hill’s values for British plants [45] for species lacking values in the former work. Based on our understanding of the natural history of *Dianthus arenarius*, we approximated its values (L = 8, R = 7, and N = 1). The Ellenberg values for individual species were weighted with their abundance in each plot to form one community-aggregated trait value for each plot.

### Statistical analyses

The index F_dis_ suggested by Laliberté & Legendre [26] was used to calculate FD. This index can handle both continuous and categorical functional variables as well as including species abundance into the analysis. F_dis_ was calculated with the package FD [46] in R 2.11.1 for Macintosh [47]. Treatment response on FD indices and species richness was analyzed by a repeated measures ANOVA and Tukey post-hoc in SPSS 18 and data were log-transformed if needed to fit normality assumptions. Non-centred Principal Component Analysis (PCA) and Redundancy analysis (RDA) with manual forward selection and 499 permutations were conducted with Canoco for Windows 4.54 (Biometris Plant Research International, The Netherlands) for species composition and functional composition data. All multivariate analyses had samples as scaling focus and all data were log-transformed. The length of the gradient for the functional compositional data justified a method assuming linear species-environment response. For verification of PCA results, a Non-metric Multidimensional Scaling with two dimensions and Bray-Curtis dissimilarities were used. We used linear regression analysis to test if succession in treated plots leads to an increased proportion of specialist species after disturbance.

## Results

**Fig 1:**
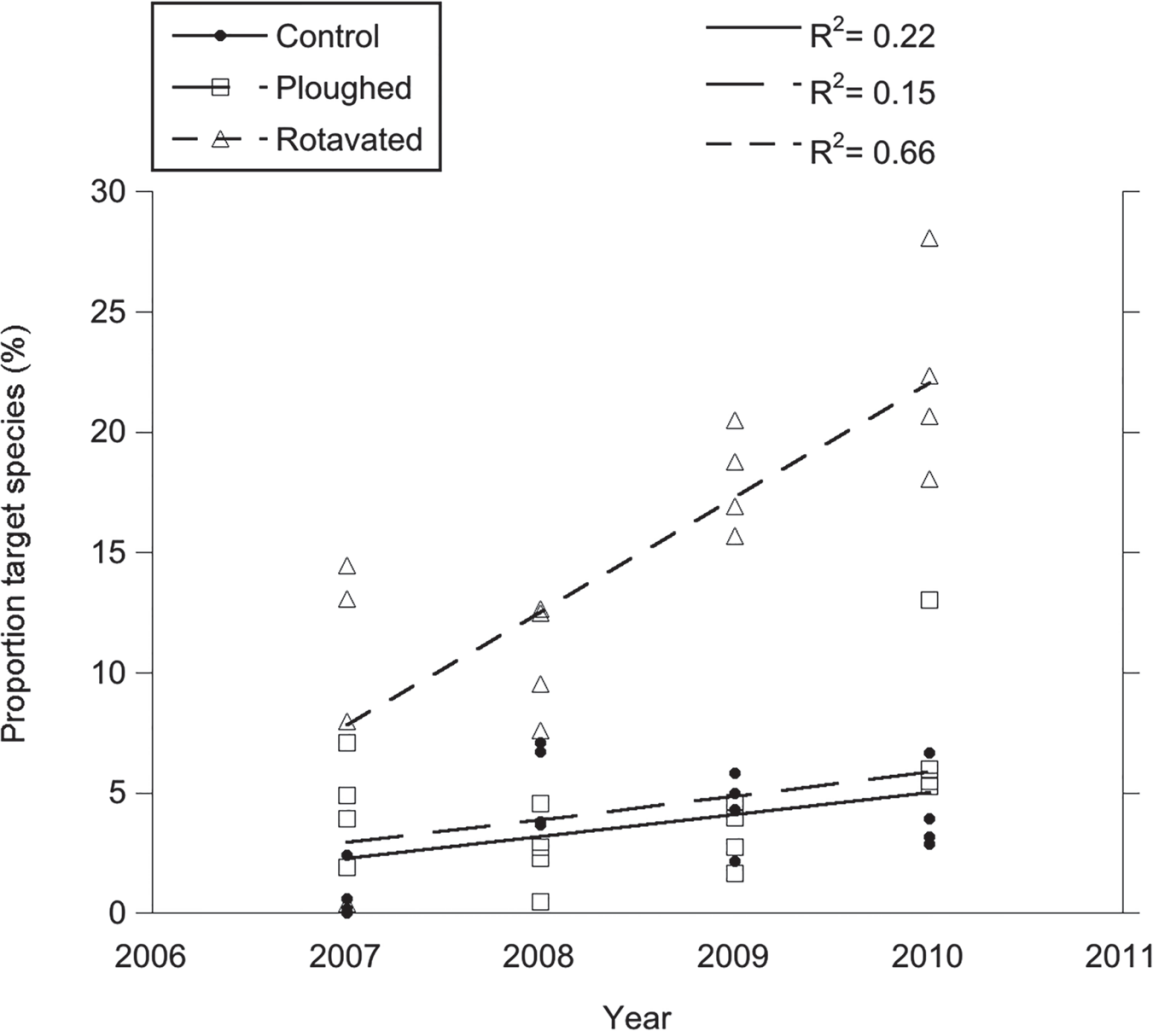
The proportion of sand steppe specialists in the vegetation during 4 years following disturbance treatment in 2006. The proportion sand steppe specialists in the target vegetation was 60%. There was a significant positive trend for rotavated plots (p<0.01), but not for controls and ploughed plots.

Sand steppe specialist species increased in rotavated plots during the four years of succession (Fig 1). However, the target vegetation had a much higher proportion of sand steppe specialist species (60%) than any of the treatment plots (Table 2). When analysing the 10 most common plant species (across the three types of treatment), including the bryophyte *Syntrichia ruraliformis*, it was found that in particular *Festuca brevipila* and *Thymus serpyllum* decreased due to disturbance, while grasses such as *Agrostis gigantea* and *Elymus repens* increased due to disturbance.

**Table 2:** Vegetation composition at the Rinkaby experimental site in 2010 (the fourth season after treatment) with the 10 most frequent species listed.

	Controls	Rotavated	Ploughed	Target
**Proportion target species (%)**				
Nationally Red-listed	-	1.2±0.93	-	18±5.2
Sand steppe specialists	4.2±0.84	22±2.1**	7.4±1.9	60±5.2
**Number of hits per plot**				
Total hits	389±18	297±13**	187±6.6***	250±2.5
Lichens	30±4.2	8.3±1.9**	0.50±0.50**	20±3.5
Bryophytes other than *Syntrichia*	86±4.3	70±4.4*	12±5.8***	12±9.5
*Syntrichia ruraliformis* *	16±3.1	60±4.7***	13±3.0	62±2.5
*Festuca brevipila*	140±22	33±3.1*	23±3.6*	0.5±0.5
*Thymus serpyllum*	33±10	2.5±1.7	5.8±3.0	13±3.0
*Galium verum*	27±4.1	9.2±3.5*	15±2.7	4.5±0.5
*Medicago sativa*	27±3.6	16±2.1*	31±3.1	22±3.5
*Plantago lanceolata*	2.2±1.1	12±2.2*	4.5±0.29	-
*Agrostis gigantea*	3.7±2.2	41±6.7***	34±8.1*	-
*Elymus repens*	0.25±0.25	9.5±3.0	21±4.6*	0.5±0.5
*Helictotrichon pubescens*	11±5.1	0.75±0.48	3.8±2.2	3.0±3.0
*Poa pratensis*	2.5±1.9	7.2±3.1	11±4.0*	-

The percentages of specialist and red-listed species was calculated from the number of the hits of such species in relation to the total number of hits, including bryophytes and lichens. Values represent means ± standard error (n = 4 experimental plots, n = 2 for target plots). The sand steppe specialists found in the study were *Alyssum alyssoides*, *Dianthus arenarius* ssp *arenarius*, *Festuca polesica*, *Koeleria glauca* and *Syntrichia ruraliformis*. Asterisks indicate significant differences between treatment plots and control.

unpaired t-test,

* = p < 0.05;

** = p < 0.01;

*** = p < 0.001.

**Fig 2:**
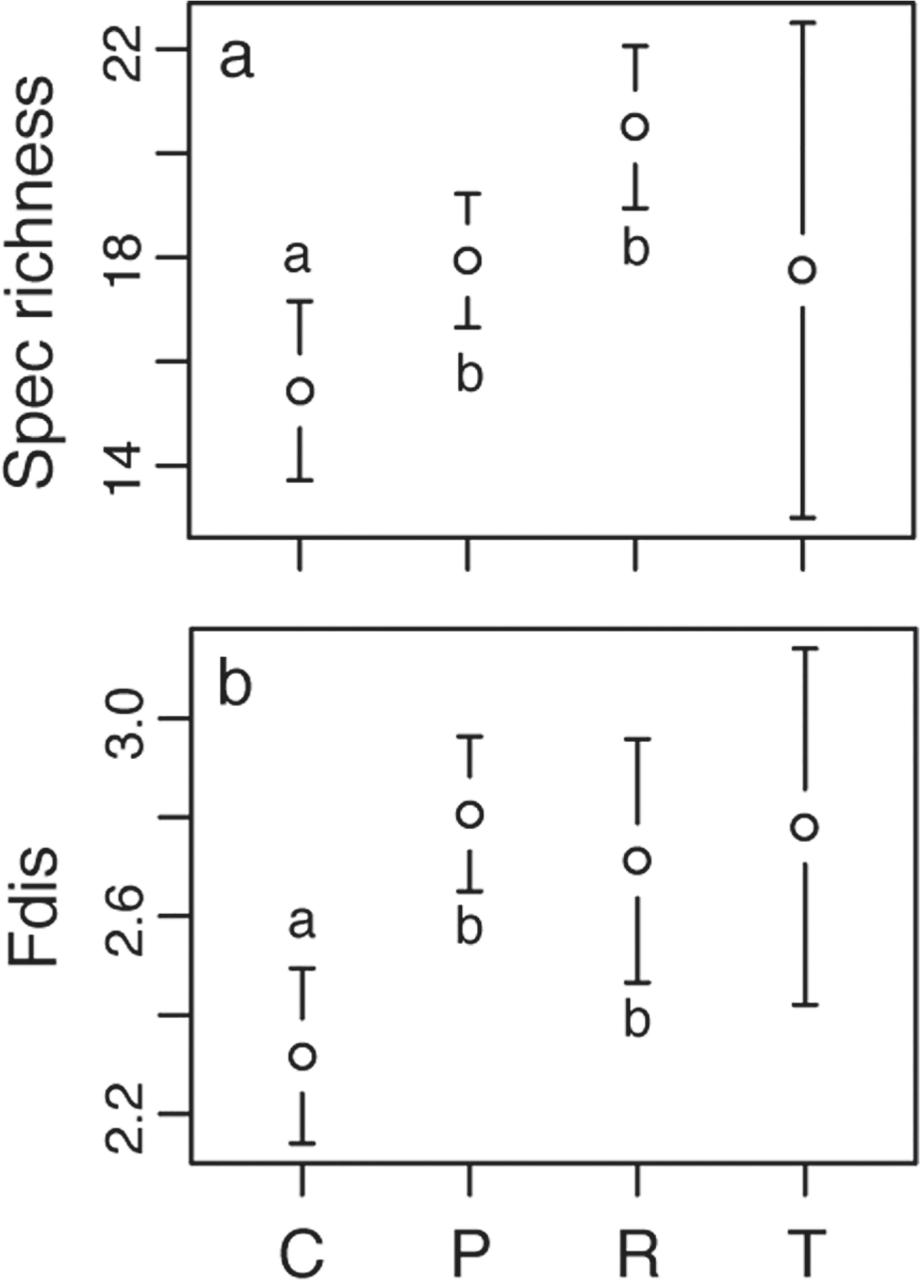
Number of species (a) and functional diversity (Fdis) (b) for each treatment. Bars show 95% CI. Letter above/below bars represent the result of statistical testing. Different letters refers to significant differences. Target was not included in the tests due to the low number of replicates. C refers to control plots, P to ploughed plots, R to rotavated plots, T to target plots. There were 4 replicates for target plots and 16 for the other treatments.

**Fig 3:**
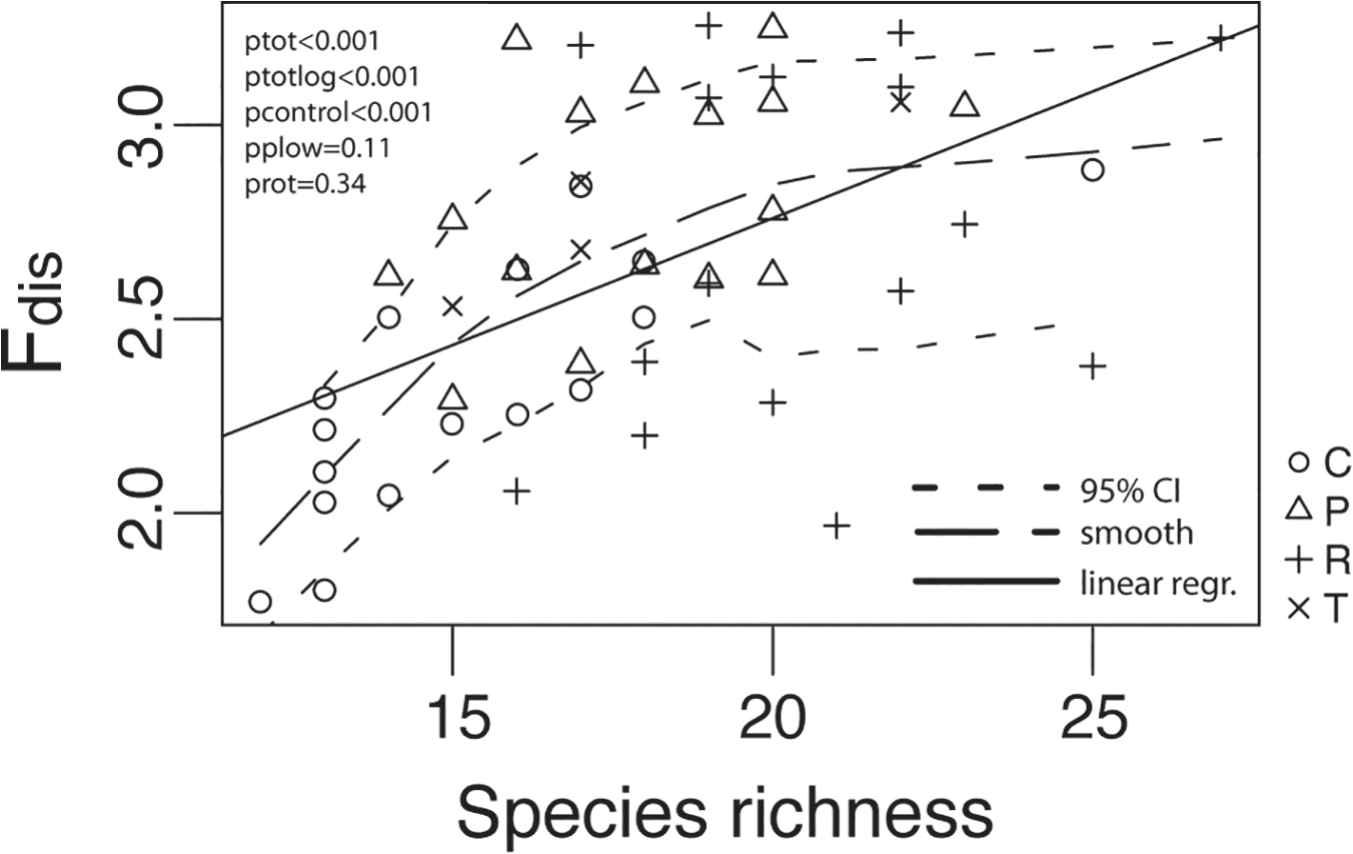
Relationship between plant species richness and functional diversity (Fdist). Linear regression is shown although a log-linear relationship had slightly higher degree of explanation because of the ease of interpretation. Broad dashed line shows smoothed relationship, and small dashed line show 95% CI to the smoothed line. C refers to control plots, P to ploughed plots, R to rotavated plots, T to target plots.

Species richness increased as an effect of ploughing and rotavation (Fig 2A). There was no effect of year on the number of species but there was a significant year × treatment interaction (Table 3), with fewer species during later years for control areas and more species in rotavated plots. The F_dis_ value differed between control plots and treated plots (Fig 2B). There was a year effect for the F_dis_ (Table 3). Treated plots tended to have greater levels of functional diversity by year 2. Overall, treated plots showed a positive trend over time with an increase in F_dis_ during the first years, and control plots had none or a negative development after the disturbance treatments. There was a significant increase in F_dis_ with species richness (Fig 3) when combining all the treatments (p<0.001, R^2^ = 0.29). However, analyzing each treatment separately, we only found this pattern for the control treatment.

**Table 3:** Results from repeated measures ANOVA of the number of species and functional diversity (Fdis) in the experimental plots.

Variable	Treatment		Posthoc (Tukey)	Time		Time*Treatment	
	F	p		F	p	F	p
*Species richness—80% dominant (log)*	8.3	<0.01	c-p p = 0.01	F = 6.3	<0.01	F = 3.6	<0.05
			c-r p = 0.02				
*Species richness Total no*. *species (log)*	17.8	0.001	c-p p = 0.03	F = 1.9 p	0.16	F = 2.7	<0.05
			c-r p = 0.001				
F_dis_	22.0	<0.001	c-p p = 0.000	F = 8.6	<0.001	F = 12.0	<0.001
			c-r p = 0.002				

Controls, rotavated and ploughed plots were included in the analysis.

**Fig 4:**
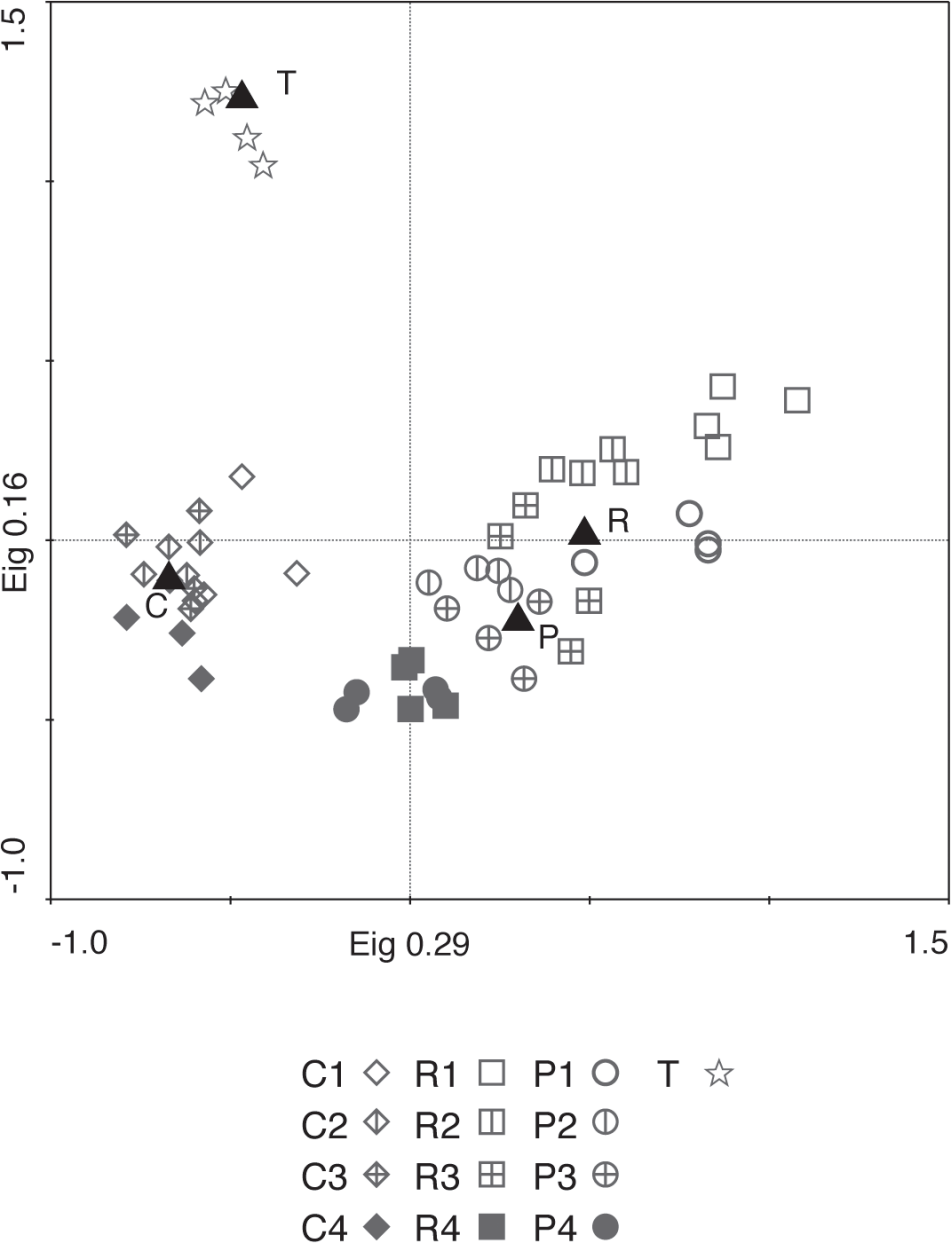
PCA plot showing the plant community composition in each of the treatments and all years. Numbers in legend refers to year after disturbance (1–4) for each treatment (C for control plots, P for ploughed plots, R for rotavated plots and T for target plots), black triangles denoted mean for each type of treatment (C, P and R) and for target plots (T).

The disturbance treatments affected the species composition of the calcareous grassland (Fig 4). The multivariate analysis distinguished the ploughed and rotavated plots from control and target plots. However, there was little or no separation between the two disturbance treatments (Fig 4), particularly in years 3 and 4. Target plots were clearly separated from both control plots and treated plots. These results remained the same irrespective of whether all species (Fig 4) or only the species from the functional analysis were included (results not shown). The Non-metric Multidimensional Scaling (not shown) gave similar result as the PCA, showing that the PCA results (Fig 4) can be considered stable. Control plots remained compositionally stable during the four years, and target areas between the two different years sampled. The treated plots showed a succession in species composition towards control plots, rather than towards target plots.

**Fig 5:**
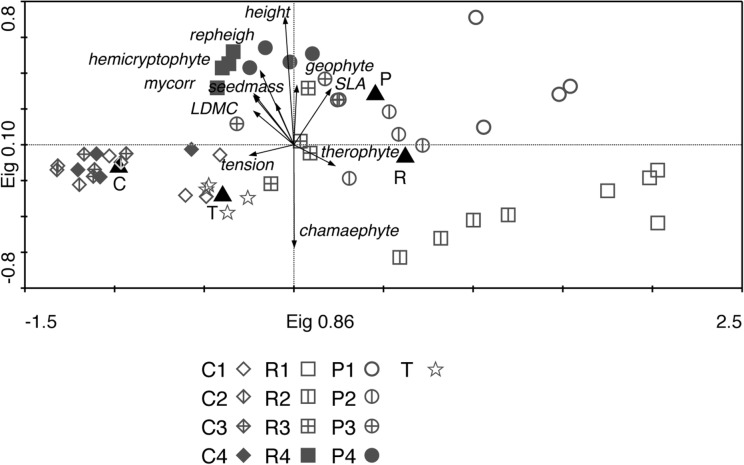
PCA plot showing the plant functional community composition based on community weighed means. Numbers in legend refers to year after disturbance (1–4) for each treatment (C for control plots, P for ploughed plots, R for rotavated plots and T for target plots), black triangles denoted mean for each type of treatment (C, P and R) and for target plots (T). Arrow text refers to the function in question. Mycorr = mycorrhizal status, repheight = reproductive height, height = plant height, tension = tensile strength.

The variation in functional trait composition was less multi-dimensional (a strong first principal component) than the species composition. In addition, there were some notable differences between the species-based PCA and the trait-based PCA (Fig 5). Similar to multivariate analyses based on species composition, we also detected differences in functional traits among treatments (Fig 5). We also detected temporal differences (axis 2) between years 1 and 2 for ploughed and rotavated areas although this axis had a relatively small eigenvalue (Fig 5). In years 3 and 4, the signatures for the two treatments overlapped (Fig 5), which indicates that functional traits of the plots converged. Although treated plots had minimal overlap with the control and target plots, the treated plots gradually became more functionally similar to the control and target plots. Canopy height, reproductive height, seed mass, mycorrhiza, SLA and LDMC were all functions that correlated to year 4 treated plots. Tensile strength, on the other hand, was positively correlated to control plots. By year four, geophytes and hemicryptophytes increased while chamaephytes decreased. Therophytes were associated mostly with treated plots in years 1 and 2.

**Fig 6:**
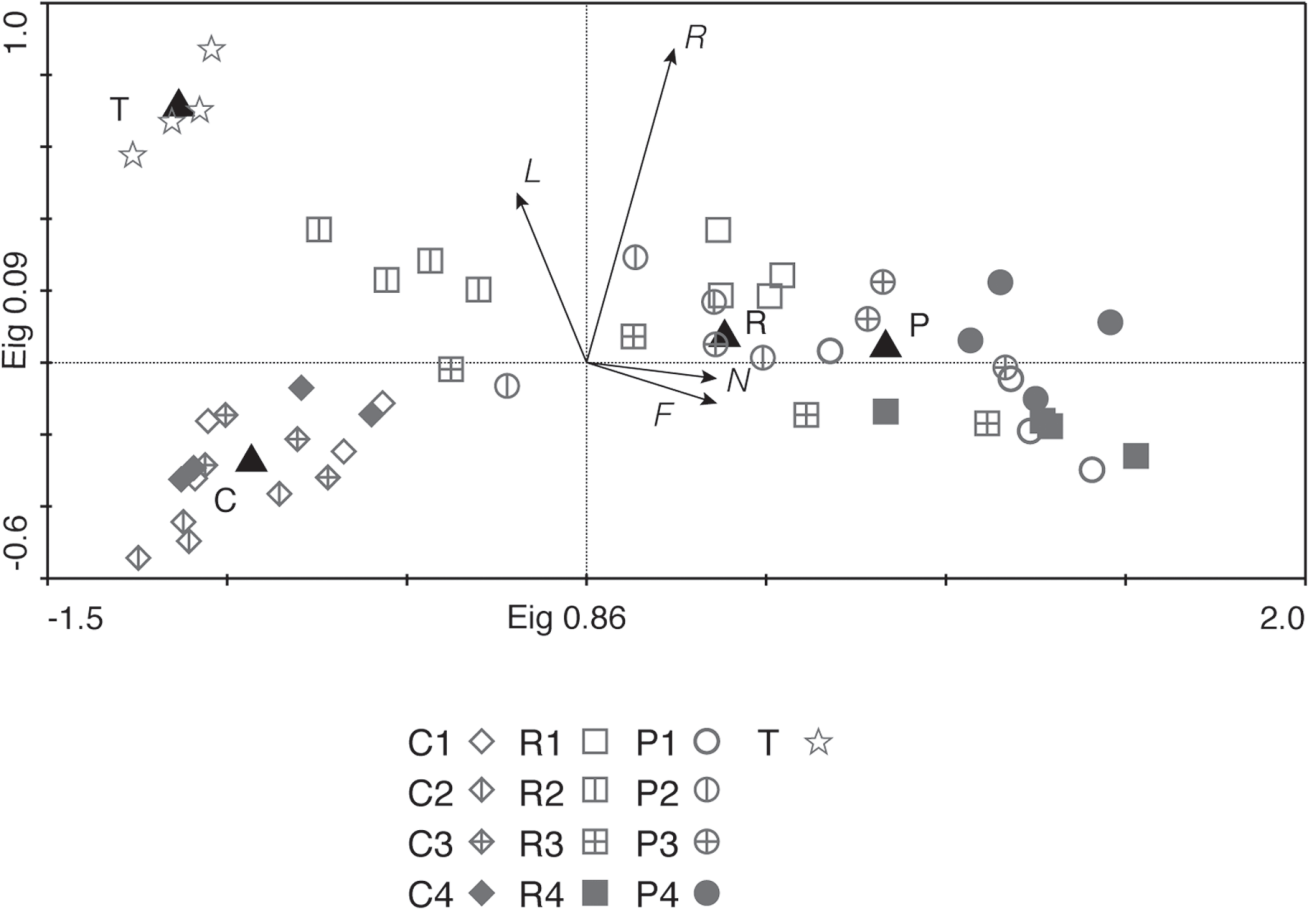
PCA plot showing the plant community composition based on community weighed means of plant species Ellenberg values. Numbers in legend refers to year after disturbance (1–4) for each treatment (C for control plots, P for ploughed plots, R for rotavated plots and T for target plots), black triangles denoted mean for each type of treatment (C, P and R) and for target plots (T). Arrow text refers to the preference for F: moisture, L: light, N: nitrogen and R: pH.

PCA on community-weighted mean Ellenberg values (Fig 6) indicated that disturbed plots developed towards a plant community with preference for higher N availability and moisture. The community means showed that targets, controls and treated plots formed three distinct groups. Target plots were separated from the study plots due to the high pH and light preference of the species in these plots. During year 1 and 2, the treated plots developed towards the target plots. During year 3 and 4, they developed in the opposite direction, away from both target and control plots.

## Discussion

We observed large effects of prescribed disturbance on species composition. While rotavation increased the proportion of habitat specialist species, showing that one of the two disturbance treatments were partly successful, none of the treatments drove species composition towards the restoration target. Instead, succession after disturbance proceeded rather rapidly towards a vegetation similar to as it was before disturbance. Others have demonstrated how difficult it can be to restore a proper disturbance regime in sandy grassland [48] and that vegetation may return to the original composition rapidly after a moderate disturbance event [49]. Our result suggests that lack of disturbance is not the only factor behind the degeneration of the characteristic sand steppe vegetation. By analyzing functional trait composition, we succeeded in getting better insight into the mechanisms driving the succession after the disturbance away from the target.

Plant succession is unpredictable, and we were therefore interested in assessing if functional diversity could be used as another tool for assessing restoration success. The inability of our disturbance treatments to shift the species composition toward the target may therefore be caused by stochastic factors during plant community assembly [15] and limited availability of propagules of the target species [50, 51]. Seed dispersal has been shown to be an important limitation in base-rich grasslands due to short-lived seed-bank [50] and short-range dispersal [52]. As suggested by Sonnier et al. [53], early succession communities are more regulated by stochastic assembly processes and dispersal limitation than by habitat filtering. Sonnier et al. [53] found weak environment-trait relationships, possibly because of dispersal and species level priority effects being more important than trait-based assembly rules. Similar to our study, they studied early successional communities and believed that this influenced their results.

The main gradient in our system was a disturbance gradient, showing that succession in our system was rapid. Height and reproductive height is considered to be related to later successional stages [43, 54, 55, 56], and SLA is considered to increase with increasing fertility [57]. Sonnier et al. [53] found that LDMC and seed mass decreases when disturbance increased. Surprisingly, height tended to increase in disturbed plots during the third and fourth year of our study. LDMC, seed mass and SLA also increased, but were not as strongly correlated to the disturbed plots. The fact that we found all these traits to be related to disturbed plots suggest that the functional discrepancy between disturbed and control/target plots is due to increased fertility following soil perturbation, at least after an initial phase where early colonizers are the only species present. Community-mean Ellenberg values also gave an indication of the environmental changes brought about by the disturbance treatments. It seems as if the disturbance treatments do, as implied above, increase the abundance of species that are associated with higher nitrogen availability than the target habitat at the end of the study. This suggests that soil disturbance increased nitrogen availability. Thus, nitrogen has an effect on the plant community, although phosphorus levels are very low and probably limiting production in the studied ecosystem [58]. This nitrogen flush could be one reason for the difference in functional composition between target plots and treatments plots during year 3 and 4, and for the increased prevalence of fertility related traits in disturbed plots seen during the later years of the study.

Disturbance may increase the occurrence of undesired species [8]. In this system we saw establishment of weedy grass species such as *Agrostis gigantea* and *Elymus repens*, which are much larger than grasses in the target plots and probably one reason for the high values for height observed in the disturbed plots. Our disturbance pushed plant communities partly in the direction of target plots in terms of functional traits, while at the same time disturbance increased nitrogen availability to levels inconsistent with successful restoration. Thus we can assume that one important factor for restoration was not fulfilled. Considering the increased variation in functional composition in disturbed plots during the first years following disturbance, the role of stochastic processes seems to have increased in disturbed areas. From the small differences in functional composition between treatments, we conclude that species functionally similar to the target species colonize after the disturbance. This may be due to abiotic conditions, such as too high P or N availability, that make the treated areas poorly suitable for establishment of the target species.

The fact that the disturbance had a positive effect on FD indicates that optimal disturbance for diversity is not prevailing in the control plots. Although we could not test for differences between target communities and our treatments, the values we have indicate that the treated plot FD is more in line with the target than the control plots. If that is the case, then we could state that the disturbance is successful in restoring FD, or at least it is a step in the right direction. Since the treatments increased both functional diversity and species richness, we conclude that in one way the treatments were successful even though the new vegetation did not resemble the target vegetation very much. The increased diversity suggests that the disturbance reduced the filtering during community assembly, resulting in more diverse communities. Similar effects have been found earlier in sand dune systems [59]. An increased functional and species diversity may have positive effects on other threatened species in the habitat. Earlier studies showed that ploughing favored beetles that are sandy grassland specialists [60], but if succession goes quickly, and not in a desirable direction, this effect may not last very long. As a part of a more general discussion regarding plant diversity, we confirmed a positive relationship between FD and species richness, which is often reported [61]. Our contrasting relationships between the treatments and the control could be because the span of species is quite small within treated plots. Sasaki et al. [62] found a positive relationship between species richness and FD with increasing grazing pressure. Mayfield et al. [63] suggests that if species richness is affected without a following effect on FD, then there is a functional redundancy in the system.

## Conclusion

We conclude that disturbance alone could not restore the desired sandy grassland community with a large proportion of specialist species. Rotavation was, however, successful in increasing the proportion of specialist species, and the analysis of functional diversity and functional composition indicated that conditions had changed in a way that could favor threatened species. A discrepancy in functional composition between target and treated plots was related to an increased average nitrogen and moisture preference of species in disturbance treatment, as shown by including analyses of Ellenberg indicator values. We conclude that a milder type of disturbance, such as rotavation, can give the best result for the vegetation, but it may have to be repeated regularly or be combined with measures that reduce nutrient availability. We recommend this method to be tested as a regular disturbance method, but the effects of repeated disturbances needs to be investigated, and this would also mimic the historical land-use in these grasslands [37]. Seeding from nearby habitats could also increase the success, once the desirable abiotic conditions have been achieved. The results show the risk of increased nutrient availability after disturbance, and that this may allow for invasion of unwanted species.
